# Supplementary material for: Monoallelic variants resulting in substitutions of MAB21L1 Arg51 Cause Aniridia and microphthalmia
Source: PLoS One. 2022 Nov 22;17(11):e0268149. doi: 10.1371/journal.pone.0268149 (PMC9681113; doi:10.1371/journal.pone.0268149)
Supplement: S1 Fig — (A) Schematic to illustrate the CRISPR-Cas9 sgRNA guide sequences and their relative locations to the Arginine 51 encoding region of the Mab21l1 locus.(B) Sanger sequencing chromatogram of PCR performed using genomic DNA prepared from a gene edited mouse. The Mab21l1 p.Arg51Leu mutation was introduced (highlighted region), along with the silent substitutions in the flanking regions (red asterisks), which were specific to the repair template. (DOCX) [file pone.0268149.s001.docx]

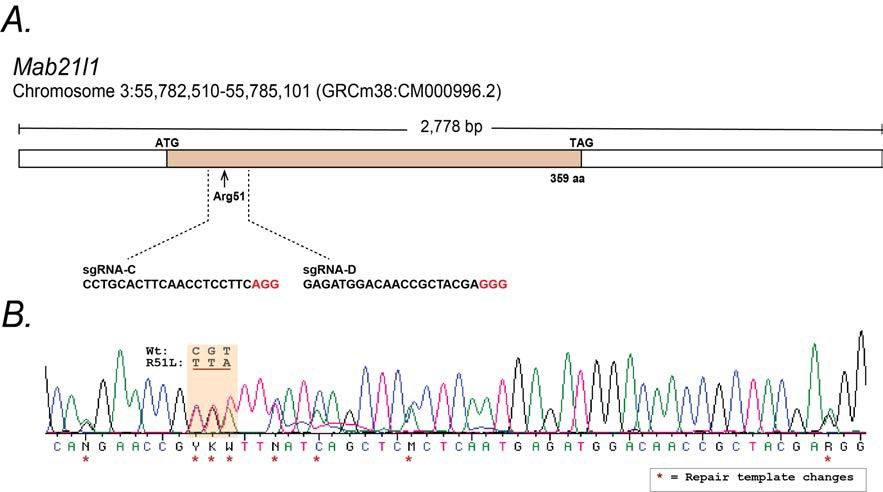


**S1 Fig: *Mab21l1* CRIPSR design. (*A*)** Schematic to illustrate the CRISPR-Cas9 sgRNA guide sequences and their relative locations to the Arginine 51 encoding region of the *Mab21l1* locus.**(*B*)** Sanger sequencing chromatogram of PCR performed using genomic DNA prepared from a gene edited mouse. The *Mab21l1* p.Arg51Leu mutation was introduced (highlighted region), along with the silent substitutions in the flanking regions (red asterisks), which were specific to the repair template.
