# Supplementary material for: Monoallelic variants resulting in substitutions of MAB21L1 Arg51 Cause Aniridia and microphthalmia
Source: PLoS One. 2022 Nov 22;17(11):e0268149. doi: 10.1371/journal.pone.0268149 (PMC9681113; doi:10.1371/journal.pone.0268149)
Supplement: S2 Fig — An allelic series of MAB21L1 heterozygous variants at position c.152G was identified in a total of five probands: two familial cases with c.152G>A (p.(Arg51Gln), chromatograms in orange shaded box), two sporadic cases with de novo inheritance of either the recurrent variant c.152G>T (p.(Arg51Leu), upper chromatogram in green shaded box) or the novel variant c.152G>C (p.(Arg51Pro), chromatogram in yellow shaded box), and one familial case with unknown genotypic inheritance of the recurrent variant c.152G>T (p.(Arg51Leu), lower chromatogram in green shaded box). Additionally, a sporadic case with unknown genotypic inheritance was heterozygous for the novel variant c.155T>G (p.(Phe52Cys), chromatogram in pink shaded box) in the adjacent 3’ codon. The chromatogram for each proband is shown, with the Family ID and pedigree case ID detailed to the right. Sanger sequencing was used to screen for and/or validate the variant in each proband, and to test all of the available relatives (data not shown), which established segregation with the phenotype. The schematic (upper right) illustrates the highly specific positioning of the four variants identified. Nucleotide and amino acid numbering is based on GenBank: NM_005584.5 and GenPept: NP_005575.1, respectively. (DOCX) [file pone.0268149.s002.docx]

**
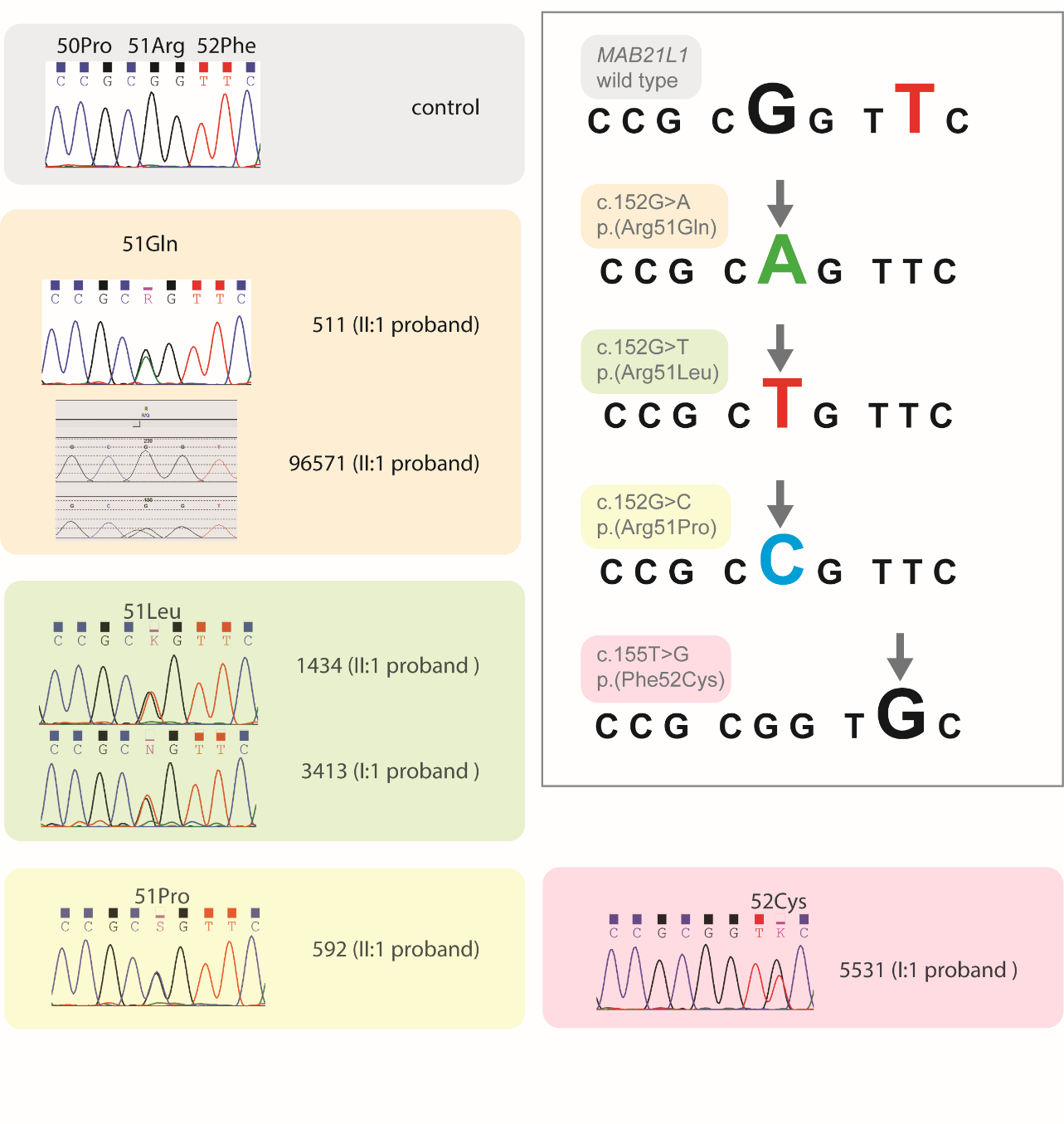
**

**S2 Fig: Sequences of highly specific *MAB21L1* heterozygous variants associated with microphthalmia and/or aniridia.** An allelic series of *MAB21L1* heterozygous variants at position c.152G was identified in a total of five probands: two familial cases with c.152G>A (p.(Arg51Gln), chromatograms in orange shaded box), two sporadic cases with *de novo* inheritance of either the recurrent variant c.152G>T (p.(Arg51Leu), upper chromatogram in green shaded box) or the novel variant c.152G>C (p.(Arg51Pro), chromatogram in yellow shaded box), and one familial case with unknown genotypic inheritance of the recurrent variant c.152G>T (p.(Arg51Leu), lower chromatogram in green shaded box). Additionally, a sporadic case with unknown genotypic inheritance was heterozygous for the novel variant c.155T>G (p.(Phe52Cys), chromatogram in pink shaded box) in the adjacent 3' codon. The chromatogram for each proband is shown, with the Family ID and pedigree case ID detailed to the right. Sanger sequencing was used to screen for and/or validate the variant in each proband, and to test all of the available relatives (data not shown), which established segregation with the phenotype. The schematic (upper right) illustrates the highly specific positioning of the four variants identified. Nucleotide and amino acid numbering is based on GenBank: NM_005584.5 and GenPept: NP_005575.1, respectively.
