# Supplementary material for: Monoallelic variants resulting in substitutions of MAB21L1 Arg51 Cause Aniridia and microphthalmia
Source: PLoS One. 2022 Nov 22;17(11):e0268149. doi: 10.1371/journal.pone.0268149 (PMC9681113; doi:10.1371/journal.pone.0268149)
Supplement: S3 Fig — Schematic representations of the linear form of MAB21L1 (blue filled bar) and MAB21L2 (purple filled bar) are shown, with the first and final amino acids numbered for each protein. For both MAB21L1 and MAB21L2 the linear positions of all published pathogenic variants are detailed on each cognate protein schematic, with the dominant heterozygous variants shown above and the recessive biallelic variants shown below. The MAB21L1 variants identified in this study are all dominantly inherited and are shown in red text. Abbreviations: dn, de novo. Nucleotide and amino acid numbering are based on GenBank NM_005584.5 and GenPept NP_005575.1, respectively. (DOCX) [file pone.0268149.s003.docx]

Arg51Pro *dn* Arg51Leu *dn* Arg51Leu Arg51Gln Arg51Gln

dominant

Arg51Gln Phe52Cys

359

**MAB21L1**

encoded by a single exon gene

1

recessive

Pro95Argfs*47 Gln233Pro Tyr280*

Cys246Leufs*18

Glu281Aspfs*20 Arg287Glufs*14

Arg51Gly Arg51His Arg51Cys *dn*

dominant

Glu49Lys Arg51Cys *dn*

359

**MAB21L2**

encoded by a single exon gene

1

Arg247Gln

**S3 Fig: Dominant and recessive variants of MAB21L1 and MAB21L2.** Schematic representations of the linear form of MAB21L1 (blue filled bar) and MAB21L2 (purple filled bar) are shown, with the first and final amino acids numbered for each protein. For both MAB21L1 and MAB21L2 the linear positions of all published pathogenic variants are detailed on each cognate protein schematic, with the dominant heterozygous variants shown above and the recessive biallelic variants shown below. The MAB21L1 variants identified in this study are all dominantly inherited and are shown in red text. Abbreviations: *dn*, de novo. Nucleotide and amino acid numbering are based on GenBank NM_005584.5 and GenPept NP_005575.1, respectively.

recessive
