# Supplementary material for: Monoallelic variants resulting in substitutions of MAB21L1 Arg51 Cause Aniridia and microphthalmia
Source: PLoS One. 2022 Nov 22;17(11):e0268149. doi: 10.1371/journal.pone.0268149 (PMC9681113; doi:10.1371/journal.pone.0268149)
Supplement: S4 Fig — Western blot analysis of the anti-TBL1XR1 IP using the anti-GFP antibody was unable to detect interaction with wild-type or mutant forms of MAB21L1.TBL1XR1 was detected in all the pull down used as control for pull down experiment. (DOCX) [file pone.0268149.s004.docx]

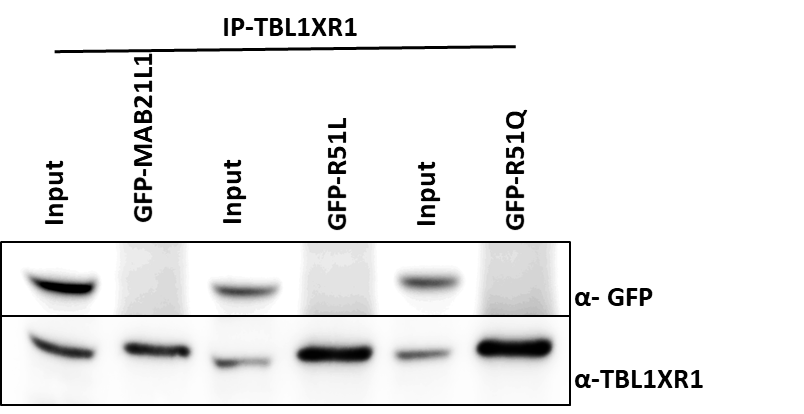


**S4 Fig:** **Reciprocal IP using TBL1XR1 antibody**. Western blot analysis of the anti-TBL1XR1 IP using the anti-GFP antibody was unable to detect interaction with wild-type or mutant forms of MAB21L1.TBL1XR1 was detected in all the pull down used as control for pull down experiment.
