# Supplementary material for: Monoallelic variants resulting in substitutions of MAB21L1 Arg51 Cause Aniridia and microphthalmia
Source: PLoS One. 2022 Nov 22;17(11):e0268149. doi: 10.1371/journal.pone.0268149 (PMC9681113; doi:10.1371/journal.pone.0268149)
Supplement: S5 Fig — A. Graphs of the log-transformed quantitative mass spectroscopy results of biological triplicates of immunoprecipitates of control (GFP), tagged wild-type (WT) and mutant MAB21L1 (R51L and R51Q) from HEK293 cells which identified as single wild-type specific interactor (TBL1XR1) which is a component of the NCor complex. Two other subunits of the NCor complex (NCOR & HDAC3) are shown for comparison. B. Graphs of the three mutant specific interactors (GALNT2, LRRFIP1, MSI2/Musashi-2) and /Musashi-1, a close homolog of MSI2, which shows interaction with all forms of MAB21L1. (DOCX) [file pone.0268149.s005.docx]

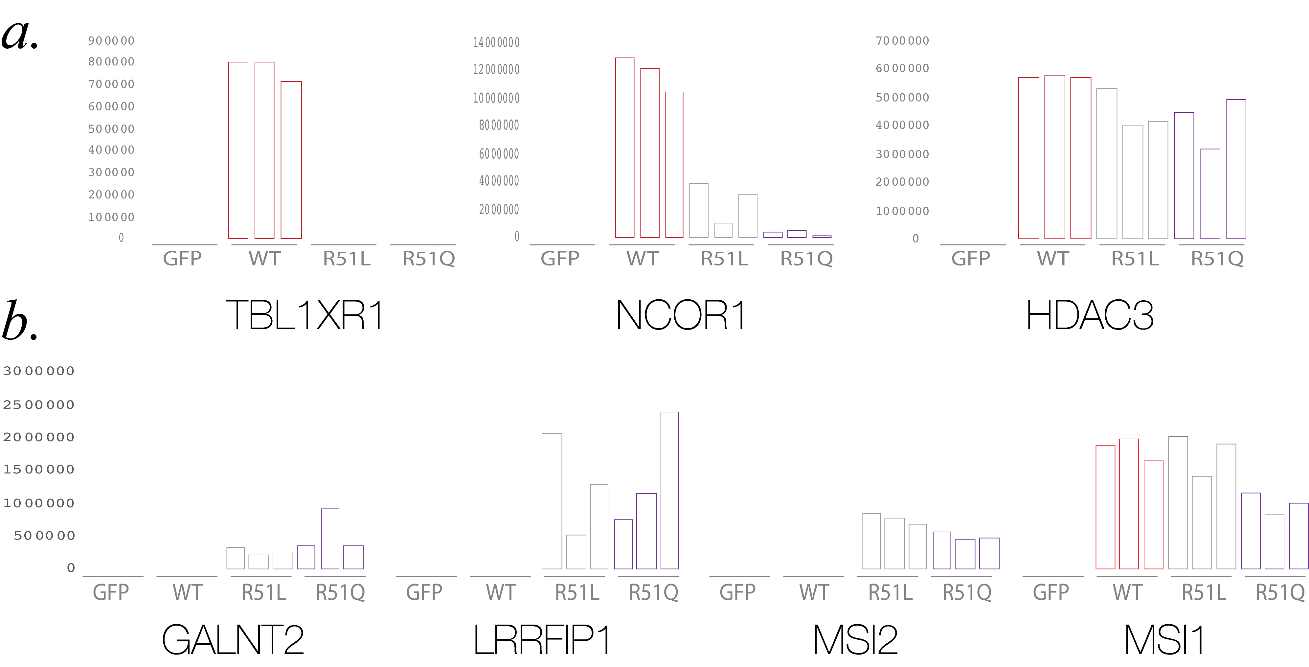


**S5 Fig: Mutant and WT-specific protein-protein interactions from IP-MS**

A. Graphs of the log-transformed quantitative mass spectroscopy results of biological triplicates of immunoprecipitates of control (GFP), tagged wild-type (WT) and mutant MAB21L1 (R51L and R51Q) from HEK293 cells which identified as single wild-type specific interactor (TBL1XR1) which is a component of the NCor complex. Two other subunits of the NCor complex (NCOR & HDAC3) are shown for comparison. B. Graphs of the three mutant specific interactors (GALNT2, LRRFIP1, MSI2/Musashi-2) and /Musashi-1, a close homolog of MSI2, which shows interaction with all forms of MAB21L1.
