# Supplementary figures and images for: Monoallelic variants resulting in substitutions of MAB21L1 Arg51 Cause Aniridia and microphthalmia
Source: PLoS One. 2022 Nov 22;17(11):e0268149. doi: 10.1371/journal.pone.0268149 (PMC9681113; doi:10.1371/journal.pone.0268149)

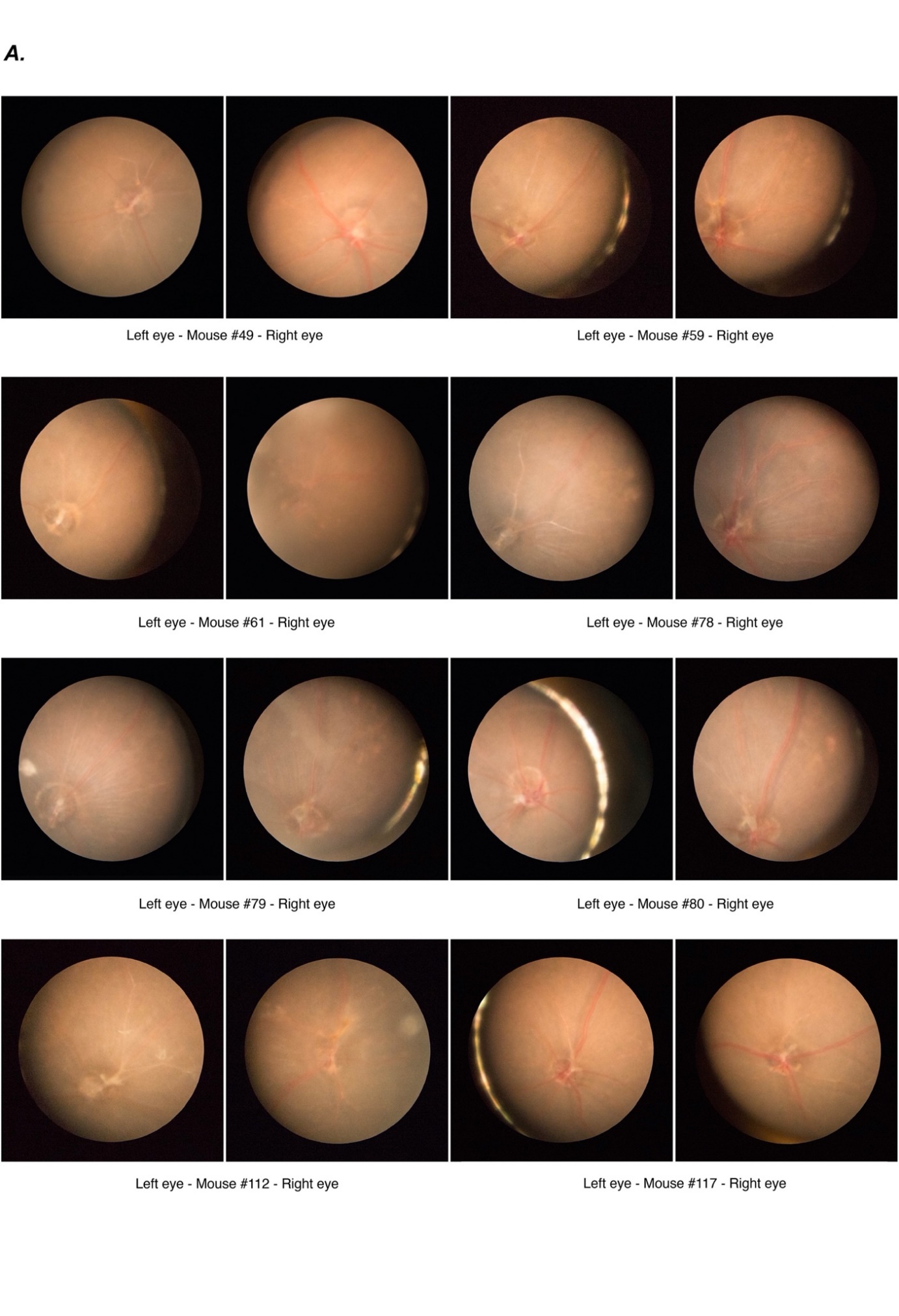

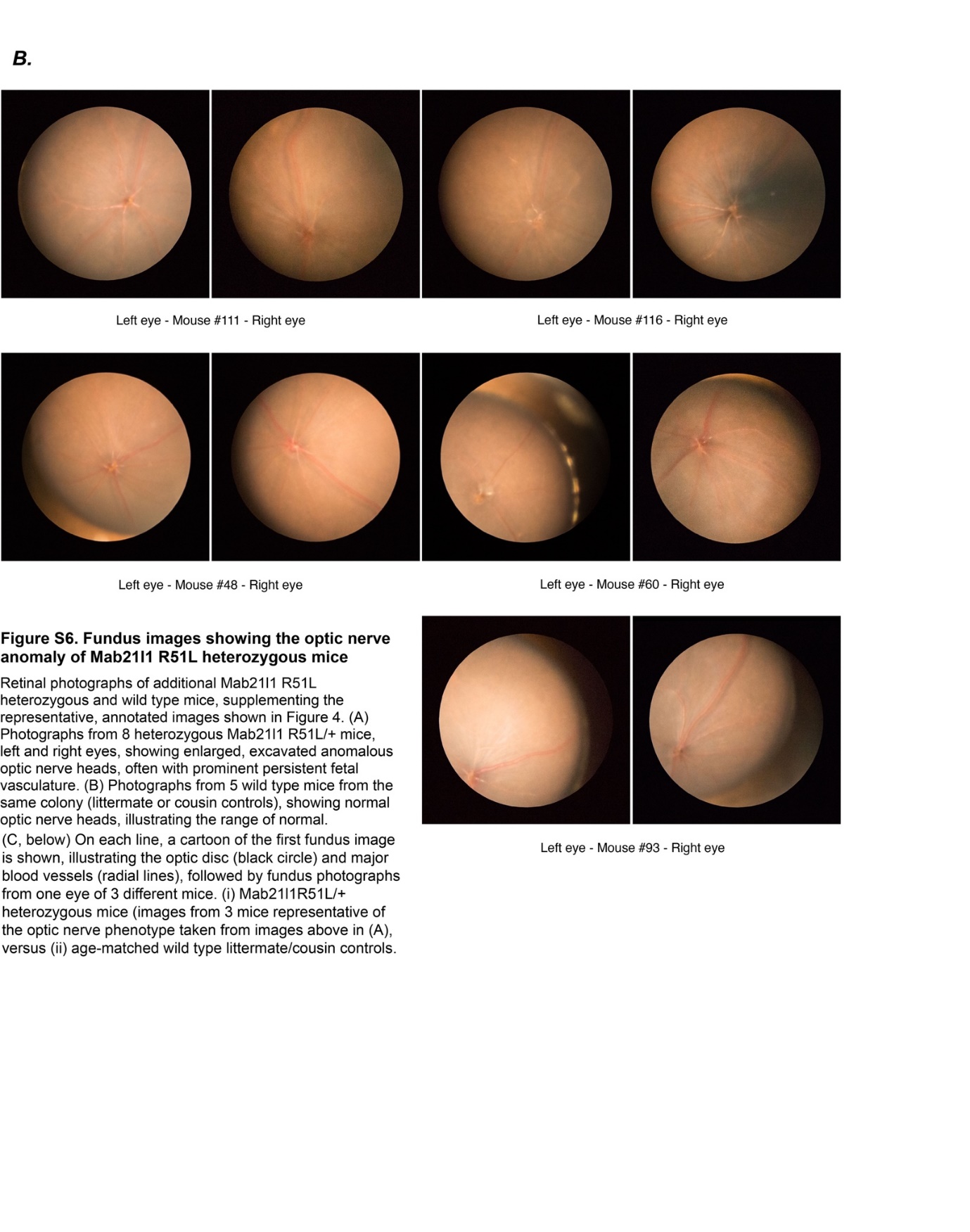


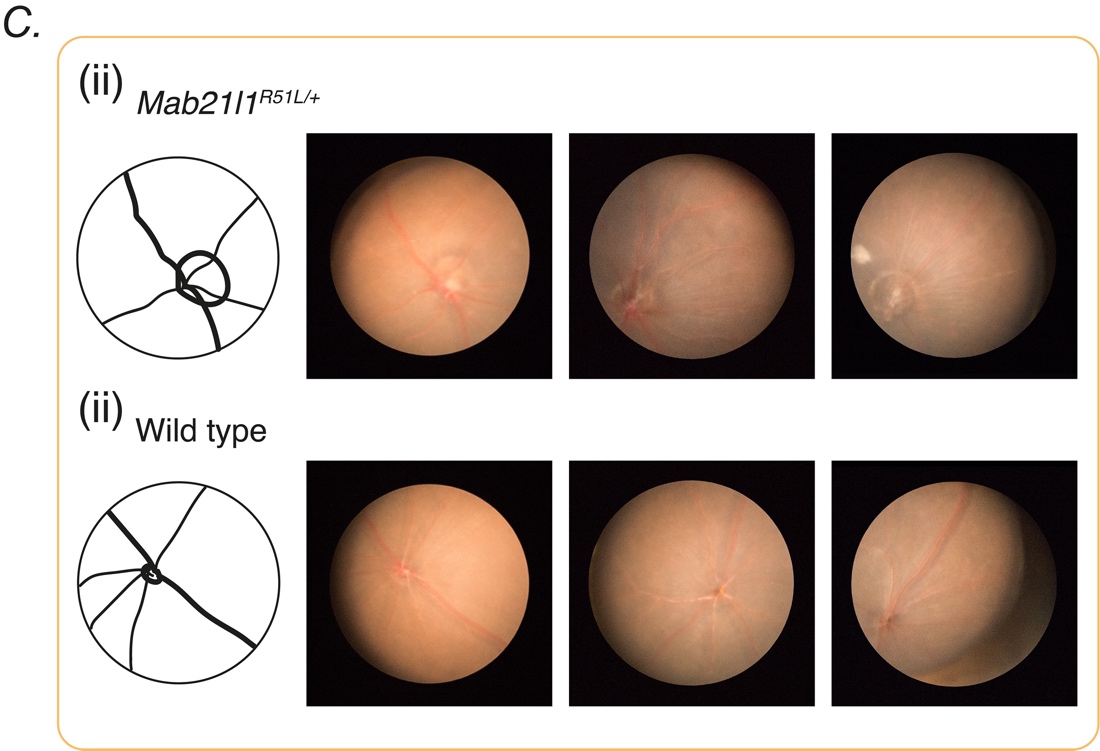

Supplement: S6 Fig — Retinal photographs of additional Mab21l1 R51L heterozygous and wild type mice, supplementing the representative, annotated images shown in Fig 4. (A) Photographs from 8 heterozygous Mab21l1 R51L/+ mice, left and right eyes, showing eniarged, excavated anomalous optic nerve heads, often with prominent persistent fetal vasculature. (B) Photographs from 5 wild type mice from the same colony (littermate or cousin controls), showing normal optic nerve heads, illustrating the range of normal. (C, below) On each line, a cartoon of the first fundus image is shown, illustrating the optic disc (black circle) and major blood vessels (radial lines), followed by fundus photographs from one eye of 3 different mice. (i) Mab21l1R51L/+ heterozygous mice (images from 3 mice representative of the optic nerve phenotype taken from images above in (A), versus (ii) age-matched wild type littermate/cousin controls. (DOCX) [file pone.0268149.s006.docx]

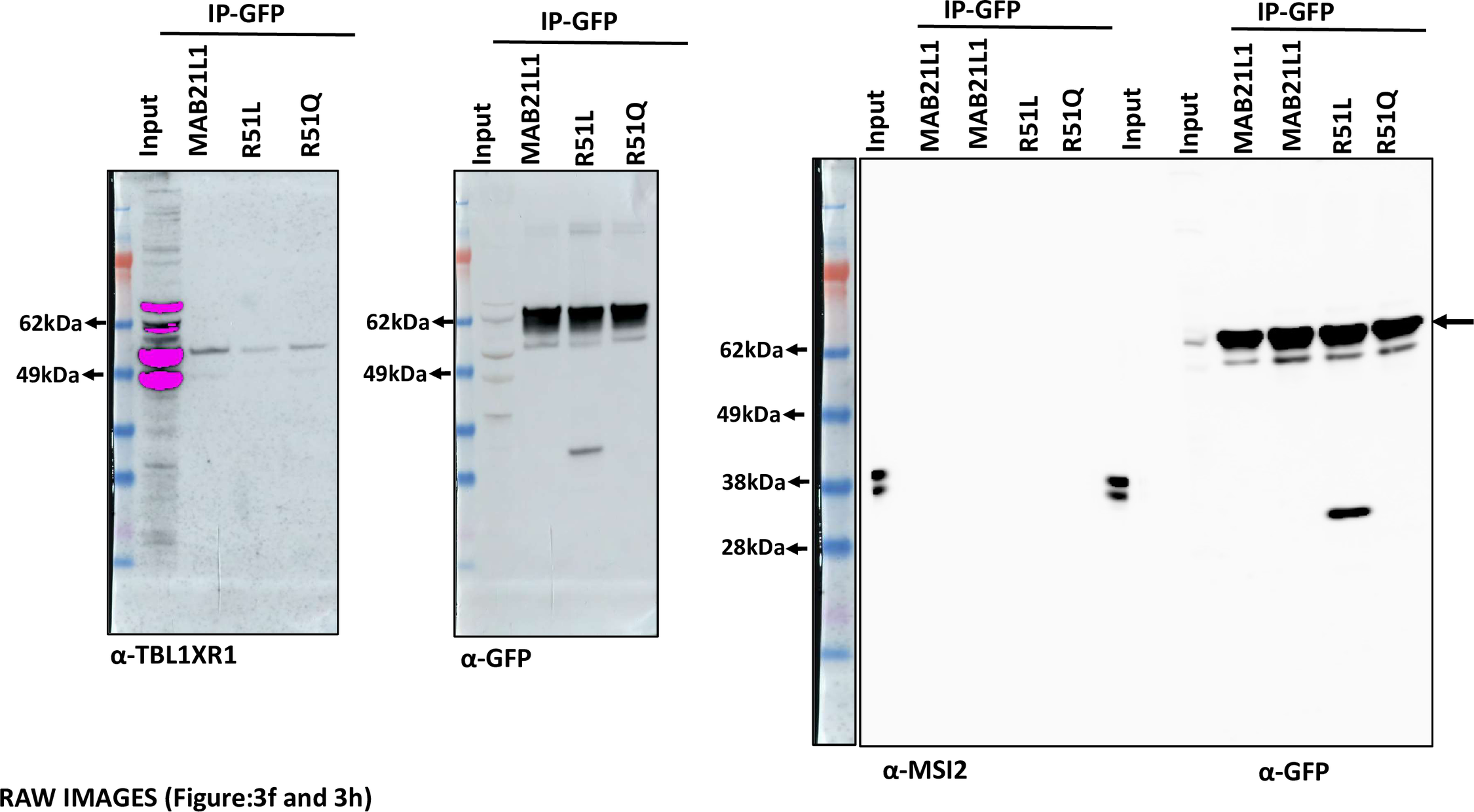

Supplement: S1 Raw images — (TIF) [file pone.0268149.s015.tif]
