# Supplementary material for: Monoallelic variants resulting in substitutions of MAB21L1 Arg51 Cause Aniridia and microphthalmia
Source: PLoS One. 2022 Nov 22;17(11):e0268149. doi: 10.1371/journal.pone.0268149 (PMC9681113; doi:10.1371/journal.pone.0268149)
Supplement: S7 Fig — A. Phylogenetic tree of the 11 human mab-21 paralogs and protein alignment (B) and genomic organisation (C) of MAB21L1, MAB21L2 and mab-21. The alignment and phylogenetic tree were generated using MUSCLE https://www.ebi.ac.uk/Tools/msa/muscle/ (DOCX) [file pone.0268149.s007.docx]

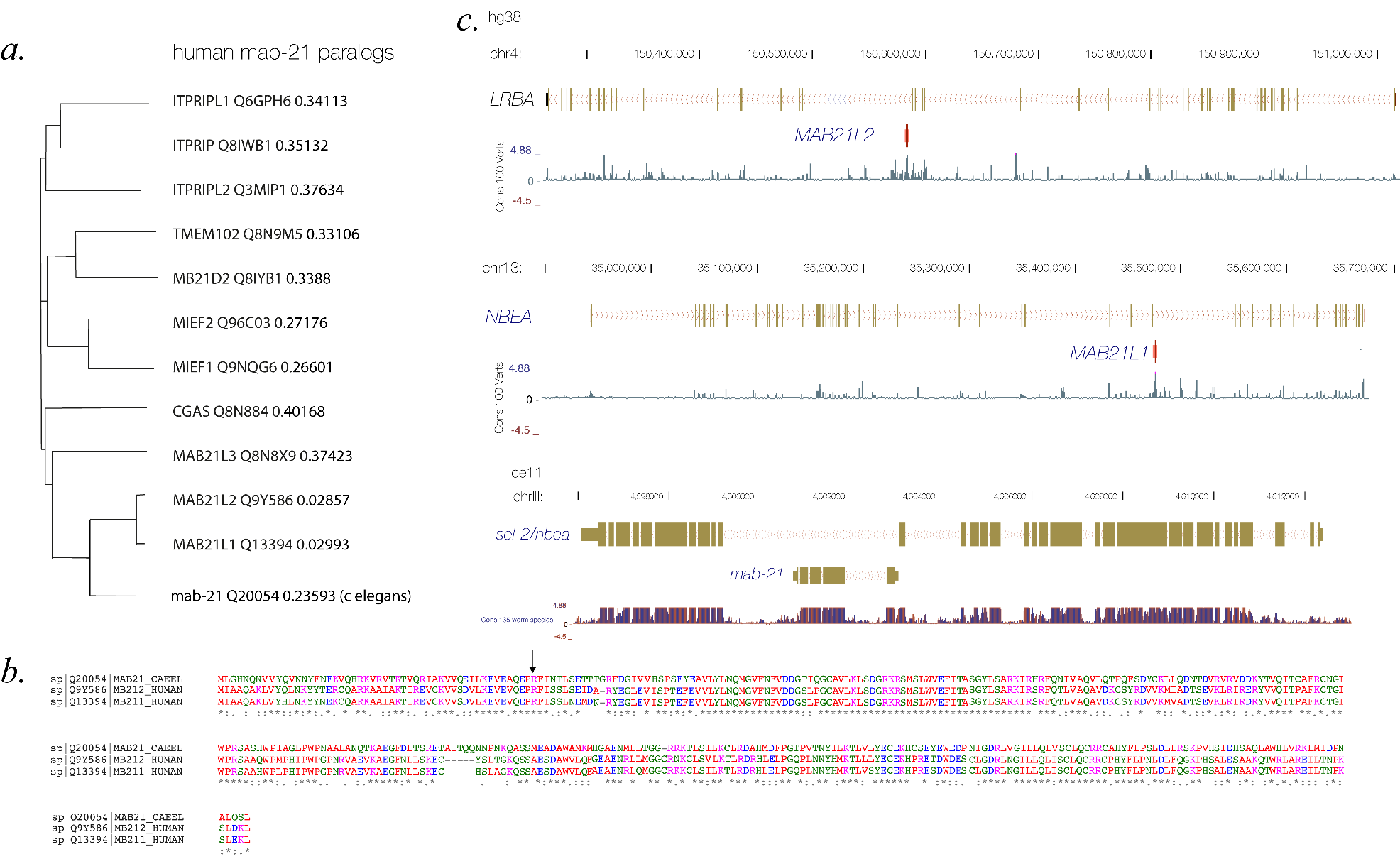


### S7 Fig: Human mab-21 paralogs: peptide sequence and genomic features

A. Phylogenetic tree of the 11 human mab-21 paralogs and protein alignment (B) and genomic organisation (C) of MAB21L1, MAB21L2 and mab-21. The alignment and phylogenetic tree were generated using MUSCLE [https://www.ebi.ac.uk/Tools/msa/muscle/](http://www.ebi.ac.uk/Tools/msa/muscle/)s
