# Supplementary material for: Monoallelic variants resulting in substitutions of MAB21L1 Arg51 Cause Aniridia and microphthalmia
Source: PLoS One. 2022 Nov 22;17(11):e0268149. doi: 10.1371/journal.pone.0268149 (PMC9681113; doi:10.1371/journal.pone.0268149)
Supplement: S1 File — (DOCX) [file pone.0268149.s008.docx]

### Supplemental Clinical Descriptions

###### Family 96571

**c.152G>A, p.(Arg51Gln) paternal mosaicism**

Two male siblings, aged 7 and 2, with non-syndromic microphthalmia/anophthalmia. The elder sibling has very severe right microphthalmia, and severe left microphthalmia with an opaque cornea, precluding a view of the internal ocular structures. Visual acuity is perception of light in the left eye only; no perception of light in the right. The younger sibling has bilateral microphthalmia and nystagmus. The right eye has an opaque cornea, again precluding visualization of internal ocular structures. The left eye has foveal hypoplasia and an anomalous optic nerve. There are no signs of cataract or glaucoma in this eye. Visual acuity is light perception in both eyes. Brain MRI, renal ultrasound and echocardiogram were normal in the older sibling, and not requested in the younger. The parents were unrelated, unaffected and had normal eyes. Testing of genomic DNA samples from both parents identified gonosomal mosaicism (at a level of ~27%) in the father.

###### Family 511

**c.152G>A, p.(Arg51Gln) familial**

A 48 year old male diagnosed with bilateral aniridia soon after birth. Both eyes are significantly microphthalmic, with a horizontal corneal diameter of 6mm in the left eye. The right eye is phthisical for unknown reasons with no view of the fundus. The left eye has progressive pannus, corneal oedema and band keratopathy. There is no discernable iris tissue, nystagmus and left post-surgical aphakia (cataract surgery aged 25). Intraocular pressure 18mmHg. View to the left posterior pole is hazy with no clear view of the fovea, but the disc appears anomalous and excavated, with a small uveal coloboma below it.

Both of his children (13 year old son and 11 year old daughter) are affected. Both children have profound bilateral aniridia, microcornea (6mm), nystagmus and gray, hypoplastic optic discs. The son has a right inferotemporal choroidal coloboma, and a small left coloboma temporal to the disc. The daughter has microphthalmia and a small left nasal choroidal coloboma; she has bilateral cataract with lenticonus in the right, and an unstable lens in the left.

His deceased parents both had ocular phenotypes: the father was similarly affected with microphthalmia and aniridia, and the mother had oculocutaneous albinism. DNA samples were not available for testing from either individual.

###### Individual 1434

**c.152G>T, p.(Arg51Leu) *de novo***

A 23 year old woman with bilateral partial aniridia, microphthalmia and glaucoma (treated surgically in the left eye). She has bilateral cataract with progressive subluxation, in addition to nystagmus and foveal hypoplasia. She is a high myope with hand movement vision in both eyes. The only other feature of note is bilateral sensorineural hearing loss; a brain MRI scan was normal. The unaffected parents did not carry the variant.

###### Individual 3413 c.152G>T, p.(Arg51Leu)

An individual with bilateral aniridia. The family was lost to follow up and consequently limited phenotypic data is available.

###### Individual 592

**c.152G>C, p.(Arg51Pro) *de novo***

An 11 year old female child with a mild aniridia-spectrum disorder and high myopia. She was born by emergency Caesarean section for fetal distress, but was subsequently well with no developmental concerns. Nystagmus, high myopia (-14 dioptres) and foveal hypoplasia were diagnosed in infancy. Electrophysiology (ERG and VEP) showed some features consistent with albinism. There was no iris transillumination but the pupillary border appeared irregular and she was described as having variant aniridia. She developed cataracts later in childhood. Her parents are unaffected and unrelated, and did not carry the variant.

###### Family 5531

**c.155T>G, p.(Phe52Cys)**

A male child with sporadic, bilateral partial aniridia and microphthalmia. The family was lost to follow- up and consequently limited phenotypic data was available.
