# Supplementary material for: Monoallelic variants resulting in substitutions of MAB21L1 Arg51 Cause Aniridia and microphthalmia
Source: PLoS One. 2022 Nov 22;17(11):e0268149. doi: 10.1371/journal.pone.0268149 (PMC9681113; doi:10.1371/journal.pone.0268149)
Supplement: S2 File — (DOCX) [file pone.0268149.s009.docx]

### Supplemental Materials and Methods

Cloning, protein purification and enzymatic assay

Wild-type human MAB21L1 and the mutant variant Arg51Leu were amplified from control and patient DNA respectively and cloned in frame into pGEX6P1 (GE LifeSciences). Purified protein was isolated from induced *E. coli* strain BL21-Gold(DE3) Competent Cells (Agilent) cultures by lysis in NETN buffer [20 mM Tris pH 8; 100 mM NaCl; 1 mM ethylenediaminetetraacetic acid (EDTA); 0.5% NP40, Complete Protease Inhibitor Cocktail (Roche)] and captured overnight on Glutathione beads (Amersham) followed by cleavage with PreScission Protease (Amersham) in buffer [50 mM Tris; 150 mM NaCl; 1 mM EDTA; 1 mM dithiothrietol (DTT) (pH 7.5)].Purification of human OAS1 protein was also performed by similar way and was used as positive control in the enzymatic assay.

A colorimetric method was used to quantitate the amount of pyrophosphate (PPi) product released upon completion of the enzymatic reaction as described (Meng et al., 2012). Purified protein and DNA or RNA were incubated in the presence of 20 mM Tris–HCl, 5 mM MgCl2, 1 mM DTT, and 2 mM nucleoside triphosphates (ATP, GTP, CTP, TTP and UTP) separately or equimolar mixture in a total volume of 100 μL at 37 °C, and the reaction was quenched after 30 min of incubation by the addition of 50 mM EDTA. We added 100 μL of molybdate reagent (2.5% ammonium molybdate in 2.5 MH2SO4) and 100 μL of 2-ME (0.5 M) to the quenched reaction mixture to produce molybdophosphoric acid. We immediately added 40 μL of Eikonogen reagent (0.125 g of 1-amino-2- naphthol-4-sulfonic acid, 0.125 g of sodium sulfite, and 7.325 g of sodium bisulfite to 100 mL of ddH2O) to this mixture, and the final volume was adjusted to 1 mL with ddH2O. The resulting chromophore molybdenum blue produced was then quantified at A580 nm measurement using a spectrophotometer.

Stable cell lines generation

Stable cell lines were generated by Flp recombinase–mediated integration using HEK-293-Flp-In T- REx host cells (ThermoFisher) transfected with pcDNA5/FRT/TO-EGFP (vector only or EGFP- MAB21L1 or mutant EGFP-MAB21L1) and pCAGGS-Flp using Lipofectamine 2000 (Life Technologies) according to the manufacturer’s guidelines. Transfected cells were selected using 5 μg/ml blasticidin and 400 μg/ml hygromycin and the resulting colonies were then expanded to establish independent cell lines in DMEM (modified Eagle’s medium; DMEM) supplemented with 10% fetal bovine serum, 5% L-glutamine, 5% penicillin/streptomycin antibiotics and 5 μg/ml blasticidin and 400 μg/ml hygromycin in 5% CO2 and normoxic conditions.

Subcellular fractionation and Western blotting

HEK-293-Flp-In T-REx tagged with EGFP-MAB21L1 or mutant EGFP-MAB21L1 were seeded in six well plate and protein expression was induced with 1 μg/ml tetracycline treatment for 12 hrs. Cells were harvested by trypsin-EDTA, washed by PBS and fractionated according to Andrews and Faller (Pubmed: 2041787). Incubation was performed in the presence of ice-cold hypotonic buffer (10 mM HEPES-KOH pH 7.9, 1.5 mM MgCl2, 10 mM KCl, O.5 mM DTT, 0.2 mM PMSF) and 0.6% of NP-40for 15 min followed by centrifugation at 15 000 g for 2 min at 4°C, and the supernatant was the cytoplasmic fraction. The pellet was washed once with the hypotonic buffer. Nuclear proteins were extracted by incubation on a rotating platform for 20 min in hypertonic buffer

(20 mM HEPES-KOH, pH 7.9. 25% glycerol, 420 mM NaCl, 1.5 mM MgCl2, 0.2 mM EDTA, 0.5 mM DTT, 0.2 mM PMSF) followed by centrifugation at 15 000 g for 15 min at 4°C and the supernatant was the nuclear fraction. The same proportion of both cytoplasmic and nuclear fraction from each cell types was analysed by western blot using anti-GFP antibody (sc- 8334; Santa Cruz Biotechnologies, Dallas, TX). Immunoblotting was performed according to the standard protocol. Protein extracts from Enhanced chemiluminescence reagents (Amersham) were used for antibody detection. All cell lines were checked for mycoplasma contamination prior to use.

SPARC expression

HEK-293-Flp-In T-Rex cells tagged with EGFP-MAB21L1 or mutant EGFP-MAB21L1 were seeded in six well plate and plasmid expressing human *PAX6* (gift from Dirk-Jan Kleinjan, University of Edinburgh) was transfected in Opti-MEM reduced serum media using Lipofectamine 2000 (Life Technologies) according to the manufacturer’s guidelines. Transfected and Untransfected cells were cultured for 48 hrs in culturing media supplemented with 1 μg/ml tetracycline.Total RNA was extracted from transfected and untransfected using the RNeasy kit (QIAGEN) following the manufacturer’s instructions. cDNA was synthesized from 1 μg of total RNA using the Roche First Strand Synthesis Kit. Quantitative PCR was performed using the Roche LightCycler 480 and the Roche LightCycler 480 Probes Master. All samples were analysed in triplicate. *SPARC* transcript levels were normalized to *GAPDH* levels for all experiments.

###### Mass Spectrometric Analysis:

The GFP pull down magnetics beads were digested in 100 µl of digest buffer (2 M Urea, 50 mM Tris– HCl pH 7.5, 1 mM DTT, 5 µg/ml porcine trypsin, (Promega)) alkylated with iodoacetamide, acidified and desalted using homemade C18 tips. The desalted and lyophilized peptides were resuspended in 0.1 % TFA and subjected to mass spectrometric analysis by reversed-phase nano-LC-MS/MS Mass spectrometry: 5 µl of the resuspended peptides were analysed by reversed-phase nano-LC-MS/MS using a nano-Ultimate 3000 liquid chromatography system and Lumos Fusion mass spectrometer (Thermo Fisher Scientific). Flow-rates were 400 nl/min. Peptides were loaded onto a self-packed analytical column (uChrom 1.6, 0.075 mm × 25 cm) using a 67-min gradient Buffer A, 2% acetonitrile 0.5% Acetic Acid, Buffer B, 80% acetonitrile, 0.5% Acetic Acid; (0–16 min: 2% buffer B, 16–56 min: 3– 35% buffer B, 56–62 min: 99% buffer B; 62-67 min 2% buffer B. The Lumos was operated in data- dependent mode with a 10-s dynamic exclusion. Full-scan spectra recording in the Orbitrap was in the range of m/z 350 to m/z 1,400 (resolution: 240,000; AGC: 7.5e5 ions). MS2 was performed in the ion trap, isolation window 0.7, AGC 2e4, HCD collision energy of 28, rapid scan rate, 50 ms maximum injection time and an overall cycle time of 1 s.

###### Database Search Parameters and Acceptance Criteria for Identifications

Raw data was analysed by the MaxQuant and Andromeda software package and protease was set to trypsin or trypsin Carbamylation (C) was selected as fixed modification. Variable modifications were N-terminal acetylation (protein), oxidation (M); FDR was set to 0.01. MS/MS spectra were searched against the human Uniprot database and a the MaxQuant contaminant database with a mass accuracy of 4.5 ppm (for MS) and 20 ppm or 0.5 Da (MS/MS OT or IT). Peak matching was selected and was limited to within a 0.7 min. elution window with a mass accuracy of 4.5 ppm.

### Supplemental Information: Mouse work

Generation of mouse model using CRISPR-Cas 9

To engineer the p.Arg51Leu change into mouse *Mab21l1* zygotes, the CRISPR/Cas9 system was used to introduce synthesised repair templates via homology directed repair (**S1 Fig A**). Two guide DNA sequences targeting the coding exon of *Mab21l1* (*Mab21l1_*guide_C and *Mab21l1_guide_D*,  **S1 Table** ) were designed for sgRNA synthesis. sgRNA were prepared using *in silico* transcription (HiScribe™ T7 High Yield RNA Synthesis Kit, New England Biolabs) as described previously (Ran et al., 2013b PMID: 24157548). A repair template DNA oligonucleotide was commercially synthesised (IDT technologies) to included the p.Arg51Leu change and additional conservative nucleotide changes to reduce subsequent HDR events at successfully edited loci (5´CGCCAAAACCATCCGGGAAGTCTGCAAAGTGGTCTCtGACGT***ttT****GAAGGAGGTTGAAGTGCAaG*AAC**C**G**tta**TTtATaAGCTCaCTCAATGAGATGGACAACCGCTACGAaGGCCTGGAGGTTATCTCTCCCACCGAGTTCG-3´). A complex of sgRNA, repair template and Cas9 mRNA (Tebu-bio) were then used for intra-zygote injections. Sanger sequencing of genomic DNA prepared from embryo tissue or adult tail-tips was used to indicate specific nucleotide changes at the targeted region and up to 150 bp of additional sequence in both directions. PCR and Sanger sequencing was performed with the *Mab21l1* locus-specific oligonucleotide primers **(S1 Table**). From the littered-down animals, a founder female animal was identified to be carrying the correctly gene-edited mutation (**S1 Fig B**) and was backcrossed to a wild type male from the parental strain C57Bl/6JCrl. Sanger sequencing showed 3 out of 8 of this litter were heterozygous, *Mab21l1^R51L/+^*, confirming germline transmission of the targeted mutation and the silent substitutions flanking it (**S1 Fig B**). The founders were not used for phenotyping experiments, however no overt developmental phenotype was observed.

Breeding, growth and health

The colony was principally maintained in the heterozygous state, backcrossing a male heterozygous animal with a female wild type animal from the parental strain, C57Bl/6JCrl, to minimize genetic drift. At least every 10 generations, a female heterozygous animal was crossed with a male wild type to refresh the sex chromosome. Heterozygous intercrosses were performed to produced homozygous animals. Littermates were used preferentially as experimental controls, then cousin or half-sibling controls; very occasionally, as this was a coisogenic strain, wild type animals from the parental C57BL/6J strain were used (ref Jackson Laboratory Handbook, ISBN 978-0-578-04182-7)

The mice appeared systemically healthy. Weights recorded in female mice on the day they had plugged were within the normal range for the mouse strain (24.5g +/- 2.7 SD at a mean of 22.7 weeks, n=7). The mean litter size at weaning for the whole colony, including all types of mating, was 6.7 mice/litter. The mean litter size for a cross of heterozygous (het) mice (usually cousin matings) was 5.8, which was not significantly different to heterozygous by wild type (WT) crosses, 7.4 (p=0.015, unpaired 2-tailed *T* test, comparing the size of n=14 litters of each type). At weaning, the ratios of the offspring inheriting the gene edited locus, as observed by direct genotyping, were Mendelian (**S3 Table** , below).
