# Supplementary material for: Monoallelic variants resulting in substitutions of MAB21L1 Arg51 Cause Aniridia and microphthalmia
Source: PLoS One. 2022 Nov 22;17(11):e0268149. doi: 10.1371/journal.pone.0268149 (PMC9681113; doi:10.1371/journal.pone.0268149)
Supplement: S5 File — (DOCX) [file pone.0268149.s012.docx]

## **Web resources**

DAVID Pathway analysis <https://david.ncifcrf.gov>

UCSC Genome Browser <http://genome.ucsc.edu>

OMIM <https://www.ncbi.nlm.nih.gov/omim/>

Bioconductor <https://bioconductor.org>
