# Supplementary material for: Monoallelic variants resulting in substitutions of MAB21L1 Arg51 Cause Aniridia and microphthalmia
Source: PLoS One. 2022 Nov 22;17(11):e0268149. doi: 10.1371/journal.pone.0268149 (PMC9681113; doi:10.1371/journal.pone.0268149)
Supplement: S7 File — S1 Table. Oligonucleotides used in the MAB21L1/Mab21l1 study: Sequence and protocol details. Underlined sequence denotes universal tags with no homology to MAB21L1. Further details of the biological relatedness microsatellite PCR protocol are available at https://www.faa.gov/data_research/research/med_humanfacs/oamtechreports/2000s/media/200614.pdf. S2 Table. MAB21L1 variant nomenclature validation. (https://variantvalidator.org/). S3 Table. Mendelian ratios. Comparison of the observed versus expected ratios of genotypes from intercrosses of the Mab21l1 R51L line mice (n = 14 litters of each type), establishing that the observed ratios were consistent with Mendelian genetics. S4 Table. FoldX values. Molecular modelling performed using FoldX (Delgado et al., 2019) in order to assess the impact of MAB21L1 and MAB21L2 substitutions on protein stability. Nearly all the mutations are destabilizing to protein structure. (DOCX) [file pone.0268149.s014.docx]

### CONTENTS

##### Supplemental Tables

**S1 Table:** Oligonucleotides.

**S2 Table:** MAB21L1 variant nomenclature validation. **S3Table:** Mendelian ratios of *Mab21l1^R51L/+^* mouse litters. **S4 Table:** FoldX values for molecular modelling.

**S1 Table: Oligonucleotides used in the *MAB21L1*/*Mab21l1* study: sequence and protocol details.** Underlined sequence denotes universal tags with no homology to *MAB21L1*. Further details of the biological relatedness microsatellite PCR protocol are available at https://[www.faa.gov/data_research/research/med_humanfacs/oamtechreports/2000s/media/200614.pdf](http://www.faa.gov/data_research/research/med_humanfacs/oamtechreports/2000s/media/200614.pdf)

| **Name** | **Sequence 5’ to 3’** | **Application** |
| --- | --- | --- |
| MAB21L1_a1_F | GTAGCGCGACGGCCAGTGAAGTTGGCTCCAGCTCTAG | NM_005584 *MAB21L1* exon1 amplicon1 PCR from human total genomic DNA |
| MAB21L1_a1_R | CAGGGCGCAGCGATGACTATCTCGGATTCTCAGTTTCAC |  |
| MAB21L1_a2_F | GTAGCGCGACGGCCAGTGCTACCTCTCGGCGCGCA | NM_005584 *MAB21L1* exon1 amplicon2 PCR from human total genomic DNA |
| MAB21L1_a2_R | CAGGGCGCAGCGATGACTTCACACTCGTAGGAAACCAG |  |
| MAB21L1_a3_F | GTAGCGCGACGGCCAGTGAAAGAAGTGCCTCTCCATCC | NM_005584 *MAB21L1* exon1 amplicon3 PCR from human total genomic DNA |
| MAB21L1_a3_Rb | CAGGGCGCAGCGATGACTTGTTTGCACTGCGGAGTGG |  |
| MAB21L1_haplo1_F | ATTTCCAAAGGCAGGGTAGG | NM_004734 *DCLK1* intron3 Microsatellite PCR from human total genomic DNA: hg19_simpleRepeat_trf 19.50XGT chr13:36533910-36534101 |
| MAB21L1_ haplo1_R | CCCAGGACAGGAATGTAAGC |  |
| MAB21L1_ haplo2_F | GCCTGCTCACTGTTGTTTGA | NM_015678 *NBEA* intron47 Microsatellite PCR from human total genomic DNA: hg19_simpleRepeat_trf 22.00XCA chr13:36169059-36169276 |
| MAB21L1_ haplo2_R | AGGGCATCGGTTATCAGAGA |  |
| MAB21L1_haplo3_F | TTGCTAGCCATTTTGTCCAT | NM_015678 *NBEA* intron41 Microsatellite PCR from human total genomic DNA: hg19_simpleRepeat_trf 20.50XTG chr13:36058316-36058510 |
| MAB21L1_haplo3_R | TGCAGGTTAAGGTTGCAAAA |  |
| MAB21L1_5’UTR_b_F | GAACGGCTGGGCTCAAAG |  |

| MAB21L1_5’UTR_b_R | AGAAACGGGCCGCAACAC | NM_015678 *NBEA* intron41, NM_005584 *MAB21L1*  exon1 (5’UTR) Microsatellite PCR from human total genomic DNA: hg19_19.66xCAG chr13: chr13:36050366- 36050526 |
| --- | --- | --- |
| MAB21L1_haplo5_F | TTGCCAGTGTTATTTGATGATTG | NM_015678 NBEA intron37 Microsatellite PCR from human total genomic DNA: hg19_simpleRepeat_trf 22.50XGT chr13:35924784-35925001 |
| MAB21L1_haplo5_R | GGGGGATAGAAAAATTCCTCA |  |
| MAB21L1_haplo6_F | TAAGTTGAGTCCGGGAATGG | NM_015678 *NBEA* intron1 Microsatellite PCR from human total genomic DNA: hg19_simpleRepeat_trf 19.50XAT chr13:35584799-35584991 |
| MAB21L1_haplo6_R | TTTGGCTCCAGTCATCTTCC |  |
| D5S818_F | GGT GAT TTT CCT CCT TGG TAT CC | Biological relatedness microsatellite PCR from human total genomic DNA: marker 1 from PCR duplex set 1 |
| D5S818_R | AGC CAC AGT TTA CAA CAT TTG TAT CT |  |
| D16S539_F | GGG GGT CTA AGA GCT TGT AAA AAG | Biological relatedness microsatellite PCR from human total genomic DNA: marker 2 from PCR duplex set 1 |
| D16S539_R | GTT TGT GTG TGC ATC TGT AAG CAT GTA TC |  |
| D3S1358_F | ACT GCA GTC CAA TCT GGG T | Biological relatedness microsatellite PCR from human total genomic DNA: marker 1 from PCR duplex set 2 |
| D3S1358_R | ATG AAA TCA ACA GAG GCT TGC |  |
| Amelogenin_F | ACC TCA TCC TGG GCA CCC TGG TT | Biological relatedness microsatellite PCR from human total genomic DNA: marker 2 from PCR duplex set 2 (XY/XX determination) |
| Amelogenin_R | AGG CTT GAG GCC AAC CAT CAG |  |
| Vwa_F | GCC CTA GTG GAT GAT AAG AAT AAT CAG TAT GTG | Biological relatedness microsatellite PCR from human total genomic DNA: marker 1 from PCR duplex set 3 |
| Vwa_R | GGA CAG ATG ATA AAT ACA TAG GAT GGA TGG |  |
| D7S820_F | ATG TTG GTC AGG CTG ACT ATG | Biological relatedness microsatellite PCR from human total genomic DNA: marker 2 from PCR duplex set 3 |
| D7S820_R | GAT TCC ACA TTT ATC CTC ATT GAC |  |
| D13S317_F | ACA GAA GTC TGG GAT GTG GAG GA |  |

| D13S317_R | GGC AGC CCA AAA AGA CAG A | Biological relatedness microsatellite PCR from human total genomic DNA: marker 1 from PCR duplex set 4 |
| --- | --- | --- |
| TPOX_F | ACT GGC ACA GAA CAG GCA CTT AGG | Biological relatedness microsatellite PCR from human total genomic DNA: marker 2 from PCR duplex set 4 |
| TPOX_R | GGA GGA ACT GGG AAC CAC ACA GGT TA |  |
| *Mus_Mab21l1*_F | CAA GCT GGT CTA CCA CCT GAA | *Mab21l1* exon 1 PCR from mouse genomic DNA |
| *Mus_Mab21l1*_R | TAA ACA CCC CCA TCT GGT TC |  |
| *Mab21l1*_guide_C | CCT GCA CTT CAA CCT CCT TC | Guide DNA sequences targeting the coding exon of  *Mab21l1* used in CRISPR-Cas9 mouse genome editing |
| *Mab21l1*_guide_D | GAG ATG GAC AAC CGC TAC GA |  |
| *Mus_Mab21l1*_F | CAA GCT GGT CTA CCA CCT GAA | *Mab21l1* exon 1 PCR from mouse genomic DNA |
| *Mus_Mab21l1*_R | TAA ACA CCC CCA TCT GGT TC |  |
| *Mab21l1*_guide_C | CCT GCA CTT CAA CCT CCT TC | Guide DNA sequences targeting the coding exon of  *Mab21l1* used in CRISPR-Cas9 mouse genome editing |
| *Mab21l1*_guide_D | GAG ATG GAC AAC CGC TAC GA |  |
| GFP-MAB21L1-F | ATGATTGCGGCCCAGGCCAAGC | Making GFP fusion constructs and protein expression constructs |
| GFP-MAB21L1-R | AATCATCCTCTAAAGTTTTTCC |  |
| *SPARC*-qPCR-F | GGTGGAAGTAGGAGAATTTGATG | Gene expression analysis (UPL-6) |
| *SPARC*-qPCR-R | TGGTTCTGGCAGGGATTT |  |
| *GAPDH*-qPCR-F | CTCTGCTCCTCCTGTTCGAC | Gene expression analysis (UPL-60) |
| *GAPDH*-qPCR-R | ACGACCAAATCCGTTGACTC |  |

### S2 Table: MAB21L1 variant nomenclature validation

#### (https://variantvalidator.org/)

| **submitted_variant** | **gene_symbol** | **transcript_description** | | **HGVS_transcript_variant** | | **HGVS_predicted_protein_consequence_tlr** | |
| --- | --- | --- | --- | --- | --- | --- | --- |
| NM_005584.4:c.152G>A | MAB21L1 | Homo sapiens mab-21 like 1 (MAB21L1), mRNA | | NM_005584.4:c.152G>A | | NP_005575.1:p.(Arg51Gln) | |
| NM_005584.4:c.152G>C | MAB21L1 | Homo sapiens mab-21 like 1 (MAB21L1), mRNA | | NM_005584.4:c.152G>C | | NP_005575.1:p.(Arg51Pro) | |
| NM_005584.4:c.152G>T | MAB21L1 | Homo sapiens mab-21 like 1 (MAB21L1), mRNA | | NM_005584.4:c.152G>T | | NP_005575.1:p.(Arg51Leu) | |
| NM_005584.4:c.155T>G | MAB21L1 | Homo sapiens mab-21 like 1 (MAB21L1), mRNA | | NM_005584.4:c.155T>G | | NP_005575.1:p.(Phe52Cys) | |
| **HGVS_predicted_protein_consequence_slr** | | **HGVS_Genomic_Description_GRCh38** | **GRCh38_CHR** | | **GRCh38_POS** | **GRCh38_REF** | **GRCh38_ALT** |
| NP_005575.1:p.(R51Q) | | NC_000013.11:g.35475987C>T | 13 | | 35475987 | C | T |
| NP_005575.1:p.(R51P) | | NC_000013.11:g.35475987C>G | 13 | | 35475987 | C | G |
| NP_005575.1:p.(R51L) | | NC_000013.11:g.35475987C>A | 13 | | 35475987 | C | A |
| NP_005575.1:p.(F52C) | | NC_000013.11:g.35475984A>C | 13 | | 35475984 | A | C |

| **HGVS_LRG_variant** | **validation_warnings** | **HGVS_RefSeqGene_variant** |
| --- | --- | --- |
| LRG_602:g.538701C>T | The current status of LRG_602 is pending therefore changes may be made to the LRG reference sequence | NG_028156.1:g.538701C>T |
| LRG_602:g.538701C>G | The current status of LRG_602 is pending therefore changes may be made to the LRG reference sequence | NG_028156.1:g.538701C>G |
| LRG_602:g.538701C>A | The current status of LRG_602 is pending therefore changes may be made to the LRG reference sequence | NG_028156.1:g.538701C>A |
| LRG_602:g.538698A>C | The current status of LRG_602 is pending therefore changes may be made to the LRG reference sequence | NG_028156.1:g.538698A>C |

*[Table split over 3 lines and GRCh37 output omitted for clarity.]*

**Metadata**

variantvalidator_version v0.2.5 variantvalidator_hgvs_version 1.1.3 uta_schema uta_20180821

seqrepo_db 21/08/2018

Mab21l1^+/+^ Mab21l1^R51L/+^ Mab21l1^R51L/R51L^

| **Het x het cross** |  | | |
| --- | --- | --- | --- |
| Observed | 16 | 44 | 14 |
| Expected (1:2:1) | 18.50 | 37 | 18.50 |

*p=0.252, chi square with 2 degrees of freedom, pooled numbers from n=14 litters*

| **Het x wt cross** |  | |
| --- | --- | --- |
| Observed | 48 | 36 |
| Expected (1:1) | 42 | 42 |

*p=0.190, chi square with 1 degree of freedom, pooled numbers from n=14 litters*

**S3 Table: Mendelian ratios.** Comparison of the observed versus expected ratios of genotypes from intercrosses of the *Mab21l1* R51L line mice (n=14 litters of each type), establishing that the observed ratios were consistent with Mendelian genetics.

| **Mutation** | **Mean ΔΔG (kcal/mol)** | **Standard deviation** |
| --- | --- | --- |
| Q233P | 7.65775 | 0.0604902 |
| R51Q | 5.51197 | 0.387943 |
| F52C | 5.34457 | 0.0648261 |
| R51G | 5.13351 | 0.176716 |
| R51C | 4.59367 | 0.380072 |
| R51H | 4.52398 | 0.413263 |
| R51L | 3.78305 | 0.248232 |
| R51P | 3.57492 | 0.309426 |
| E49K | 1.99549 | 0.51072 |
| R247Q | -0.647993 | 0.171486 |

**S4 Table: FoldX values.** Molecular modelling performed using FoldX (Delgado et al., 2019) in order to assess the impact of MAB21L1 and MAB21L2 substitutions on protein stability. Nearly all the mutations are destabilizing to protein structure.
